# Impact of a Six-Year Project to Enhance the Awareness of Community-Based Palliative Care on the Place of Death

**DOI:** 10.1089/jpm.2017.0696

**Published:** 2018-10-09

**Authors:** Nozomu Murakami, Kouichi Tanabe, Tatsuya Morita, Yasunaga Fujikawa, Shiro Koseki, Shinya Kajiura, Kazunori Nakajima, Ryuji Hayashi

**Affiliations:** ^1^ Department of Palliative Care Surgery, Kouseiren Takaoka Hospital, Takaoka, Toyama, Japan.; ^2^ Drug Informatics, Faculty of Pharmacy, Meijo University, Nagoya, Aichi, Japan.; ^3^ Department of Palliative and Supportive Care, Seirei Mikatahara Hospital, Hamamatsu, Shizuoka, Japan.; ^4^ A Board of Palliative Care, Saiseikai Takaoka Hospital, Takaoka, Toyama, Japan.; ^5^ Home Palliative Care Committee, Takaoka Medical Service Region, Takaoka, Toyama, Japan.; ^6^ Department of Medical Oncology, Toyama University Hospital, Toyama, Toyama, Japan.

**Keywords:** community-based palliative care, multiprofessional team care, outreach, palliative home care

## Abstract

***Object:*** To examine the clinical outcomes of a project to enhance the awareness of community-based palliative care (awareness-enhancing project), focusing on home death and care rates in communities.

***Methods:*** A single-center study on community-based intervention was conducted. The awareness-enhancing project, consisting of three intervention approaches (outreach, palliative care education for community-based medical professionals, and information-sharing tool use), was executed, and changes in the home death rate in the community were examined.

***Results:*** The home death rate markedly exceeded the national mean from 2010. In 2012–2013, it was as high as 19.9%, greater than the previous 5.9% (*p* = 0.001). Through multivariate analysis, the participation of home care physicians and visiting nurses in a palliative care education program, and patients' Palliative Prognostic Index values were identified as factors significantly influencing the home death rate.

***Conclusion:*** The three intervention approaches time dependently increased the home death rate as a clinical outcome in the community, although they targeted limited areas. These approaches may aid in increasing the number of individuals who die in their homes.

## Introduction

The number of patients who desire palliative home care has been reported as potentially large^1,2^; however, many people do not realistically consider home death in Japan without general practitioner systems.^3,4^ In such a situation, increasing the number of those who die in their homes through palliative home care is an approach to fulfill patient desires. It is also a challenge to be urgently addressed.^5^

The paucity of resources for home medicine, such as home-visit nursing facilities, is particularly marked in Toyama Prefecture, which has a lower home death rate than the national mean. However, it is difficult to increase these resources within a short period. In addition, (1) insufficient primary-care physicians well-versed in palliative care, (2) insufficient basic palliative care-related knowledge and skills of community medical staff, and (3) their widely varying specialties make information-sharing difficult. To resolve these problems as much as possible and increase the home death rate, we developed and executed a project to enhance the awareness of community-based palliative care (awareness-enhancing project), consisting of three intervention approaches: (1) outreach visits by a hospital palliative care team (PCT) to compensate for insufficient functions in the community using the program (outreach program); (2) palliative care education for community-based medical professionals using an educational program Palliative Care Emphasis Program on Symptom Management and Assessment for Continuous Medical Education (PEACE)^6,7^; and (3) using a newly developed information-sharing tool, Regional Referral Clinical Pathway for Home-based Palliative Care (RRCP-HPC).^8^ As the educational program was developed for hospital physicians not specializing in palliative care, its effects on home care physicians and other medical professionals engaged in home care have yet to be clarified.

This study examined the effects of these three intervention approaches on the home death and care rates in the community.

## Methods

### Outline

This single-center study on community-based intervention was conducted in two steps: (1) evaluating the overall effects of the awareness-enhancing project on the home death rate in the community; and (2) evaluating the effects of PEACE-based education and RRCP-HPC use on the home death and care rates in the community. The study was conducted based on a protocol approved by the Ethics Review Board of Saiseikai Takaoka Hospital.

### Development and use of the outreach program

#### Development of the outreach program

The home-visit PCT of Toyama Prefecture Saiseikai Takaoka Hospital, comprising three types of medical professionals, certified palliative care physicians, nurses certified for such care, and medical social workers, visited patient homes (outreach visits) based on the outreach program. Pharmacists and other medical professionals also participated in the team as necessary. A draft of the program was created upon deliberations among PCT members. Subsequently, a pilot version was created under the supervision of external palliative care specialists and home care physicians, and it was used for several cases to establish the final version.

Using this program, the home-visit PCT addressed issues classified into the following categories, which were not covered by home care physicians: symptom assessment, physical and mental symptom control, and advice on social/financial issues. The visiting frequency was once a week, and the main target area was the Takaoka Secondary Medical Zone in Toyama prefecture.

Furthermore, we adopted the RRCP-HPC as an information-sharing tool. This tool comprises three sheets: a symptom assessment sheet based on support team assessment scheduling, a medical records sheet, and a free description sheet with spaces to report the progress of multidisciplinary care team activities and give anticipatory instructions to manage sudden changes in each symptom. As it is considered a part of the patient-held records, this novel information-sharing tool promotes participation by both patients and their families.

### Targets

Cancer patients referred to the PCT of Toyama Prefecture Saiseikai Takaoka Hospital, and discharged home between October 2007 and October 2013 were targeted.

### Outcomes and their evaluation

To clarify the influence of the adopted PEACE on patients through community-based medical professionals, the home death rate was examined as the primary outcome. During the six-year study period, it was calculated every two years and compared with that in the Toyama Medical Zones adjacent to the targeted Takaoka Medical Zones as a control with similar background factors, such as the number of medical institutions per population. The number of cancer patients who had died at home within each period was divided by the total number of cancer deaths within the same period, using mortality-related data contained in the vital statistics published by the Ministry of Health, Labor, and Welfare.

### Provision of the PEACE-based education for medical professionals

#### Adoption of the PEACE

The PEACE, established in October 2007,^6,7^ was used to educate medical professionals in both Toyama Prefecture Saiseikai Takaoka Hospital and the Takaoka Secondary Medical Zone. All the educators who provided the program were previously certified as palliative care specialists or advisors engaged in specialized palliative care in the clinical setting and completed a two-day three-night PEACE educator training program. There were seven palliative care physicians, two palliative care surgeons, one psycho-oncologist, and one pain relief specialist. Two of the palliative care physicians were also providing home care.

### Targets

Cancer patients who had received treatment at a single community cancer center within the Takaoka Medical Zones between October 2006 and September 2013 were targeted.

### Outcomes and their evaluation

The place of death as an outcome was evaluated using multiple logistic regression analysis to investigate how the implemented PEACE has influenced patients through community-based medical professionals. Subsequently, the home care rate as another outcome, calculated using the following formula, was examined through multiple regression analysis: [(total number of days spent at home while receiving care/number of days survived after a joint conference) × 100]. In all cases, the age, sex, Palliative Prognostic Index (PPI), and performance status (PS) were used as adjustment factors for confounding variables.

### Statistical analysis

For comparison between groups, the *t* test, Mann-Whitney U test, chi-square test, and Fisher's exact test were used according to the type of data. Changes in the home death rate were compared using a generalized estimating equation (robust estimator). The significance level was set at 0.05 in all cases. To clarify the effect size, Cramer's V or Cohen's d was calculated.

All statistical procedures were performed using SPSS version 22 (IBM Japan Ltd., Tokyo).

## Results

### Effects of combined intervention, including outreach visits, on the home death rate

The number of patients visited per day was 3.8 ± 1.7 (mean ± SD). The mean number of home visits per patient was 4.6 ± 6.6. Among the 522 cancer patients who died at home in the Takaoka Medical Zones during the intervention period, 71 (13.6%) had been treated with the outreach program. Optional care procedures performed by the PCT replacing home care physicians included the following: central venous port maintenance (36), continuous subcutaneous morphine administration (26), delirium management (17), expensive drug administration (12: 7 cases of continuous subcutaneous octreotide acetate administration and 5 cases of zoledronic acid administration), ascites puncture for drainage (8), and home oxygen therapy (4). In all cases, symptoms were assessed, and advice on social/financial issues was provided.

The PEACE-based education was provided once a year since September 2008, and the total number of participants was 189 as of October 2013 (91.7% among the 206 doctors working at general clinics in the Takaoka Medical Zones). Their specialties were as follows: internal medicine: 114, surgery: 42, and others: 33. There were 19 working at facilities certified as clinics supporting home care.

Similarly, the use of the RRCP-HPC was initiated in October 2011; until October 2013, the program was used in a total of 62 cases (40.5% among the 153 cancer patients who died at home in the Takaoka Medical Zones within the same period).

**FIG. 1.**
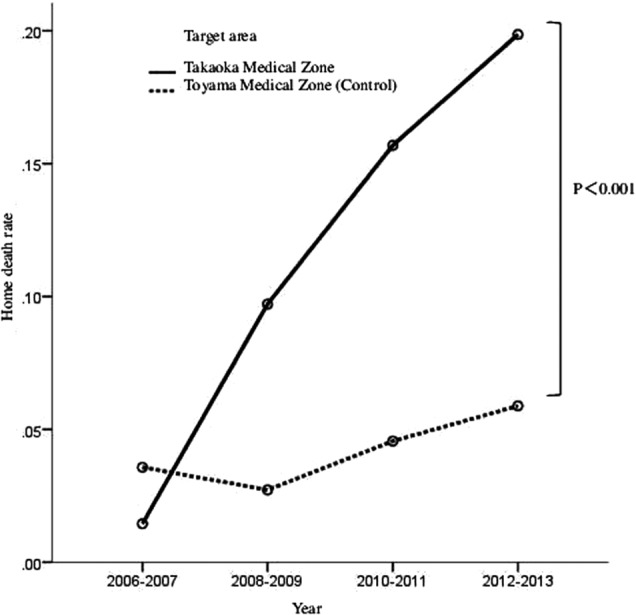
Time-dependent changes in the home death rate.

The home death rate markedly exceeded that in the Toyama medical zone (control) from 2010. In 2012–2013, it was as high as 19.9%, greater than the previous 5.9% (*p* < 0.001) (Fig. 1).

### Effects of PEACE-based education on the home death and care rates

**FIG. 2.**
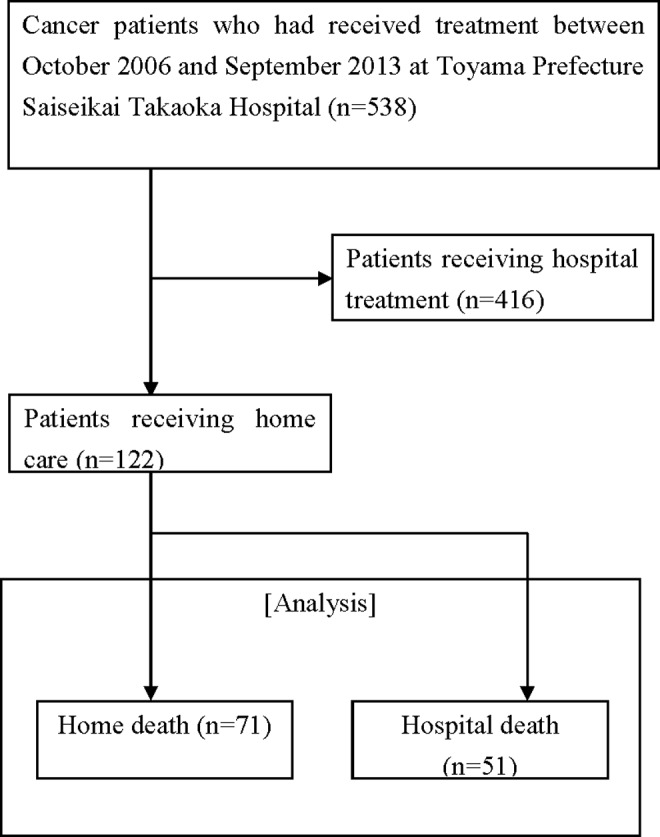
Patient classification flowchart.

Among the 538 patients registered for the study cohort, 122 (hospital death: 51; and home death: 71) who received home care were analyzed (Fig. 2).

Through univariate analysis, the participation of home care physicians and visiting nurses in the PEACE-based education, and patient PPI values were identified as factors significantly influencing the home death rate (Table 1). Multiple logistic regression analysis also confirmed the marked influences of these three factors on the home death rate (Table 2).

**Table 1.** Patient Backgrounds

*Factor*	*Hospital death group (*n* = 51)*	*Home death group (*n* = 71)*	p
Total number of days spent at home while receiving care (mean ± SD)	55.6 ± 68.2	41.4 ± 39.6	0.15
PS at the time of joint conference (*n*, 0/1/2/3/4)	0/11/17/21/2	0/10/25/28/8	0.25
PPI at the time of joint conference (mean ± SD)	3.0 ± 2.1	4.3 ± 2.5	0.003^*^
Age (years, mean ± SD)	72.2 ± 14.1	72.6 ± 13.1	0.86
Sex (*n*, female/male)	28/23	32/39	0.28
Visiting frequency (times/week, mean ± SD)	0.70 ± 0.36	0.78 ± 0.55	0.31
Participation of home care physicians in the PEACE-based education (*n*, not participated/participated)	29/22	22/49	0.004^*^
Participation of visiting nurses in the PEACE-based education (*n*, not participated/participated)	41/10	34/37	<0.001^*^
RRCP-HPC use (*n*, not used/used)	31/20	39/32	0.52
Target area (*n*, Takaoka city/others)	39/12	44/27	0.09
Caregiver (*n*, wife/others)	20/31	26/45	0.77
Opioid use (*n*, not used/used)	14/37	19/52	0.93

^*^ *p* < 0.05.

PEACE, Palliative Care Emphasis Program on Symptom Management and Assessment for Continuous Medical Education; PPI, Palliative Prognostic Index; PS, performance status; RRCP-HPC, Regional Referral Clinical Pathway for Home-based Palliative Care.

**Table 2.** Factors Influencing the Home Death Rate (Multiple Logistic Regression Analysis)

*Explanatory variable (factor)*	p	*Odds ratio*	*95% Confidence interval*
PPI at the time of joint conference	0.002	1.43	1.14–1.81
Participation of home care physicians in the PEACE-based education (reference: not participated)	0.027	2.66	1.12–6.33
Participation of visiting nurses in the PEACE-based education (reference: not participated)	<0.001	8.51	2.67–27.41

Among the age, PPI, PS, participation of home care physicians and visiting nurses in the PEACE-based education, and RRCP-HPC use as factors influencing the home care rate, RRCP-HPC use was solely extracted as such an influencing factor on multiple regression analysis (*p* = 0.047, *β* = 0.20 [95% confidence interval: 0.003–98.3]).

### Content of intervention (medical care provided for the patients)

#### Hospital death group

The physician in charge intervened through collaboration with doctors specializing in fields other than palliative care such as oncologists and surgeons. The consultation-type PCT of the study hospital performed routine monitoring and provided care on a 24-hour basis for 365 days as necessary.

#### Home death group

A home care physician based in the community was in charge of caring for each patient. The PCT of the study hospital performed monitoring (outreach) weekly and provided care on a 24-hour basis for 365 days as necessary. In addition, from October 2011, the use of the RRCP-HPC as a newly developed information-sharing tool was initiated. Using this tool, information regarding the patient was shared at a multiprofessional joint conference before, and among the physician in charge, visiting nurse, pharmacist, care manager, and hospital PCT after discharge to home; after discharge, medical professionals recorded the patient's condition on the RRCP-HPC form on every outreach visit. The patient and family also entered their comments.

#### Intervention for both groups

For both groups, palliative care was provided by multiprofessional care teams made up of physicians, nurses, pharmacists, medical social workers, and other professionals. Only the physician in charge, use of information-sharing tools, and frequency of routinely holding palliative care conferences varied between the groups.

## Discussion

This study examined the clinical outcomes of a six-year project to enhance the awareness of palliative care, consisting of three intervention approaches: performing outreach, providing palliative care education for community-based medical professionals, and effectively using the RRCP-HPC. Although they targeted limited areas of the community, these approaches were effective to time-dependently increase the home death rate as a clinical outcome. In previous studies, difficulty in appropriately performing procedures not covered by home care physicians, such as catheter management and expensive drug administration, tended to be a barrier to home care.^9,10^ Based on this, the outreach approach provided in this study may have contributed to the increase in the home death rate. Furthermore, it has been reported that once patients are hospitalized due to deterioration, the probability of discharge decreases, consequently increasing hospital deaths.^11^ Prompt consultation and management provided by palliative care specialists are needed to prevent such situations.^11^ The outreach approach may have contributed to this.

Another important point in this study is that the participation of both home care physicians and visiting nurses in the PEACE-based education was found to be an independent factor influencing the home death rate. In other countries, hypofunction, patient desire for home death, living with close relatives, and the availability of home care resources have been reported to be associated with home death, and the necessity of training physicians in private practice, in addition to organizing administrative systems for family support, has been noted.^12–14^

Highlighting the importance of palliative care education for community-based medical professionals, the results of previous studies also support the findings of our study, and they are in accordance with the current direction of such care in Japan.

As for the RRCP-HPC, its use also markedly influenced the home care rate. It is a type of patient-held record that has been reported to be useful.^15,16^ Previously, the authors conducted preliminary studies to confirm its usefulness, especially for palliative home care.^17,18^ Interestingly, in this study, this tool was found to be useful for the continuation of home care. As demonstrated in previous studies,^4,19^ home care is not more disadvantageous to end-stage cancer patients in their last days than hospital treatment, but factors explaining this have yet to be clarified. The contribution of RRCP-HPC use to the survival time also remained unclear in this study. However, the results suggest that information-sharing among medical professionals prolongs the duration of home care.

This study has some limitations: the items were retrospectively examined; it was a relatively small sample size; only patients of good physical condition might be discharged; and it was difficult to clarify whether home care physicians and visiting nurses after the PEACE-based education were engaged in community-based palliative care independently. In addition, the influence of RRCP-HPC use on the home care rate cannot be discussed as it was not measured. Furthermore, the subgroup analysis to examine the influences of PEACE only targeted patients who had received home care. As the patients themselves decided whether to receive home care or hospital treatment, there may have been a sampling bias.

## Conclusion

Outreach activities, palliative care education for home care physicians and visiting nurses, and RRCP-HPC use may aid in increasing the number of individuals who die in their homes.
